# Six Letters, and Answers to Correspondents

**Published:** 1872-07

**Authors:** 


### Our Correspondence.

### LETTERS EXPOSING HUMBUGS AND SWINDLERS,

WITH OTHER COMMUNICATIONS, OF INTEREST TO THE PEOPLE.

LATROBE, PA., May 27, 1872.

THAD S. UP DE GRAFF, M. D.

*Dear Sir*: I take pleasure in sending you a copy of Pierce's paper, and I do hope you will give him his just dues, for verily, he is as big a humbug as ever entered the field of secret medicines and nostrums. If you can find any Hygiene or Domestic Medicine in this, give him credit; but I assure you I can't see any. "When roughs fight among themselves," we may hope for good results for honest people.

W. C. COLEMAN, M. D.

—The paper sent us by Dr. Coleman, is simply an advertising sheet for "Pierce's Family Medicines." It condemns all other patent nostrums, and devotes its remaining space to extolling the wonderful medicinal excellencies of "Dr. Pierce's Family Medicines."

All we have to say about the matter is, that people *will* be humbugged, always have, and we suppose always will buy the patent nostrums which are thrust under their noses in every drug store and corner grocery throughout the land. Therefore, Dr. Pierce might as well reap a harvest from such ignoramuses, as anybody else.

---

COILA, N. Y., April 15, 1872.

EDITOR BISTOURY:

*Sir:* An error occurs in your copy of a formula sent by me to the *Boston Journal of Chemistry*, for the cure of "Poison by Ivy." If you will take the trouble to turn to the *Journal* ot September last, you will see that I did not write "White Oak," but "White Ash." Please give your compositor to understand that the two are not identical; and when you rap him over the knuckles for his blunder, do not fail to enjoin upon him the duty of making the necessary correction in your next number.

J. D. STEWART, M. D.

—The above letter explains itself. We have made the necessary correction in this number, reproducing the article referred to as it *should* be. It will be found under the head of Domestic Medicine.

---

—We are constantly in receipt of complimentary letters of the BISTOURY, from both professional and non-professional sources. It is rarely that we publish any of them, but the following one being short, to the point, and from an eminent medical man, we give it room among our budget of letters:

CEDAR KEYS, FLA., March 5, 1872.

DR. THAD S. UP DE GRAFF:

*Dear Sir:* Please enroll my name on your list of subscribers for the Bistoury. It is certainly the cheapest, and at the same time one of the most valuable medical journals of the day.

Yours, Respectfully,
GEO. A. PENNERY, M. D.

---

MOSCOW, LUZERNE CO., PA., April 17, 1872.

T. S. UP DE GRAFF, M. D.

*Dear Sir:* Your favor, with bill enclosed, received. With this I enclose one dollar, clearing myself from charges on your books, and advance pay for the current volume. Now, that matters have taken the shape I am accustomed to, I will hereafter need no hint, nor threat of a collector, to keep me in the right way. I have been a subscriber to many periodicals, for more than half a century, and was never in debt for any; always paying when I order, and not having any booked to me until I ordered. Many of your readers, no doubt, will feel greatly indebted for the information gratuitously given them in regard to the newspaper law, making it so plain that a wayfaring man, though a fool, may not again err. Wonder if your correspondent at Jamestown, N. Y., does not also feel under great obligation? Hope we may hear through the Bistoury, or some other source, if his colt yet lives; if so, and saved by any, or all of your prescriptions, I would not need any further evidence of your skill as an M. D., as well as being a profound jurist.

"TO THE POINT."—As I feel that every word in the last number under this caption, is intended as much for me as any other of your delinquent *subscribers*, and as, of course, you meant to give no offence, you will in like manner take none by reason of anything I may say.

How did I become a subscriber? I take it for granted the same as probably all of your delinquents—by having it sent them unsolicited; and having the advantage of the law protecting publishers from loss, to assure you of payment, provided the receiver of the journal did not order it discontinued.

Taking it for granted that we are the very essence of meanness, as you have made it appear, it is not always a fatal disease; as evidence of this, would you and I have survived it?

Now, that matters are in shape, and as it is supposed that we understand each other, I can hereafter peruse your valuable and entertaining paper with greater pleasure, knowing it is paid for. Very Respectfully,

C. A. H.

--The only error under which our spicy correspondent is laboring, is that of supposing that the magazine was sent him without his consent. By referring to our books, we find that he is one of a great number who had the BISTORY sent them for three years, when it was a free journal, no price being asked for it; perhaps *then* it was sent unsolicited, and only cost the receiver four cents a year for postage. When our circulation became so large that we were unable to supply the demand for the journal, and as *everybody* was likely to become subscribers to so useful a paper when nothing was asked for it, we were compelled to decline extending its circulation, or ask payment for doing so. We accordingly issued it in magazine form, and placed within it a printed circular, in which we informed our subscribers that the small sum of *fifty cents* would be charged for it in future, at the same time asking all those who declined to receive it upon such terms, to return it to us marked "refused," and with their post office address. If the journal was not so returned, we would consider the person to whom it was addressed a *bona fide* subscriber, willing to receive the journal, and to pay the sum asked for it. And that is how our friend C. A. H., together with many other delinquents, became subscribers to our journal.

We contend that there exists no where in the land, a journal possessing the merit of THE BISTOURY, for which so small a subscription price is asked. It is not our desire to force it upon any one,—our circulation is increasing as rapidly as we desire. It is *not* a money-making concern—every dollar received for it is expended in improving its usefulness. We publish it for the pleasure it affords us, and do not care a "continental" (whatever that is), whether *anybody* takes it or not.

---

ADRIAN, MICH., June 1, 1872.

DR. UP DE GRAFF:

*Sir:* I sent to Pittsburg some three months ago to Dr. J. Henry Smith, for some of Mr. Yardley's Wild Tea, to cure a cancer on my face. I paid ten dollars for a small case of it, which proved to be dried and pounded plantan leaves, such as you can find in your door yards, and which you would pay ten dollars to get rid of. It is needless to say it did my cancer no good, and that J. Henry Smith, and his mythical Chas. Yardley, are humbugs.

J. L. R.

We exposed the Yardley swindle in our last number. Read the BISTOURY, and we will keep you posted upon all new medical swindles that may arise.

---

*C. T. S., Brooklyn, N. Y.*—The back numbers of the Bistoury were sent you. If they have not yet come to hand, let us know. Better let "stomach bitters" alone, and apply to your physician for a tonic, if one is needed.

---

*E. H., Lockhart, Texas.*—The registered letter has not yet come to hand.

---

*Dr. G. D. M., Hartford, Kan.*—The blank sent you was an error of the mailing clerk. Your subscription to the Bistoury is paid up.

---

*Rev. M. W. R., Lima, N. Y.*—When you pay arrearages we will discontinue the journal, and not before.

---

*C. R., Sardinia, N. Y.*—For the pain in your joints, without knowing the cause, we would prescribe the following liniment:

Take—
Tincture of camphor,
Tincture of opium,
Tincture of capsicum,
Liquor ammonia, of each, two oz.

Mix, and rub your joints well with the mixture every night, before retiring.

---

*Dr. A. A. D., Milan, Tenn.*—You have forgotten that the Bistoury is a quarterly, and not monthly. We cannot, therefore, send you a May number.

---

*Dr. J. B., Muncie, Pa.*—Send to Geo. Tieman & Co., for their illustrated circular. You will there find what you desire. Address, 67 Chatham St., New York.

---

*Dr. W., Louisville, Ky.*—An ophthalmiscopic examination would decide the point. Take him to some ophthalmic surgeon.

*Rev. J. D., DeGraff, Ohio.*—Read an article in last number of the Bistoury, entitled Astigmatism, for the information you desire.

---

*Mrs. M., Penn Yan, N. Y.*—Write Dr. Babcock, whose address you will find in the Bistoury. His supporter is the best we know of.

---

ADRIAN, MICH., May 20, 1872.

EDITOR BISTOURY:

I send you the enclosed circular of Dr. C. M. Mack, of Indianapolis, Ind. Do you suppose he is really what he represents himself to be, as follows: "Graduate of Guy's Hospital, London, and for over two years confidential Physician to Her Majesty the Queen; and late of the Medical University of New York." You see he claims to be able to "treat the sick abroad as well as in the city." Can he do it, and would you trust him? B.

—You are evidently a new subscriber to the Bistoury, else you would have no occasion to ask such questions. Set Mack down as a humbug—you will be perfectly safe in so doing. We will wager you a cookie he never saw the inside of a medical institution, either of this or any other country. If he were ever "Confidential Physician to her Majesty the Queen," for two seconds, there would be no occasion for his locating in Indianapolis, advertising to cure all manner of diseases for the small sum of "five dollars, securely sent by post office order." Not a bit of it! He's altogether *too* liberal and too certain of his cures. Write him so, and spell his name with a J,—the M is evidenfly an error on his bill.

---

WILLIAMSPORT, PA., May 1, 1872.

EDITOR BISTOURY:

I want to tell you how provokingly I was swindled by the advertisement in *Harper's Weekly*, of the "Electro Magnetic Curling Comb."

You have seen the elegant engraving of this wonderful comb in the journal referred to.—Wasn't it taking though? I do wonder how many silly little innocents, like myself, were "taken in" by this attractive announcement? Well, I sent the necessary $1.25 to the advertiser, and in the course of two months, after having written several times for it, the wonderful comb arrived. Would you believe it?—'t was only a very diminutive, ordinary, horn comb, such as you can buy in any store for five cents. With it came directions for me to go to a tin shop and procure a piece of sheet copper, two inches long and one-eighth of an inch wide, and to then proceed as follows:

"Place the copper bar in one of the grooves that run lengthwise of the comb; place the zinc bar in the other groove; then take a strong silk thread, beginning at one end of the comb and wind once between each tooth over the bars and back of the comb, until you have passed it around 30 times, then fasten; then begin at the other end of the comb and wind as before, and the same number of times around the bars and comb. Now take the enclosed copper wire and wind as you did the thread, filling the space in the middle of the comb between the thread. You will now have a complete Electro-Magnetic Curling Comb.

Comb the hair you wish to curl briskly for two or three minutes with the Electro-Magnetic Curling Comb; then take a curling stick made of black walnut (that being the best conductor), the size you wish the curl, and with a clean hair brush wind the hair around the stick. You now will have a beautiful curl which will retain its shape and beauty, with ordinary care, from two to five days.—The Electro-Magnetic Curling Comb *must be used* ONLY on FALSE hair."

Now, isn't that sublimely ridiculous? After paying one dollar and twenty-five cents for a five cent comb, I must hunt up the necessary "Electro-Magnetic" apparatus and make the machine myself. Of course, I saw at a glance the affair was a premeditated swindle, and that there was no curling qualities in *such* a comb when completed; so, I thought I would write to the Bistoury and tell you how nicely I was swindled, hoping I might save others from the same investment. But don't, for the world, tell anybody my name; I don't like to be considered foolish, notwithstanding I feel so. Just put a blank to this communication, like this:

—— ——

The young lady tells her own story admirably. We have nothing to add, save that we think such highly respectable journals as the HARPERS publish, might find sufficiently remunerative advertisements from honest men, and abandon those of acknowledged swindlers, with which they constantly keep their advertising columns filled

